# Increase in Penicillin Non-Susceptibility in Group B Streptococci Alongside Rising Isolation Rates—Based on 24 Years of Clinical Data from a Single University Hospital

**DOI:** 10.3390/antibiotics14090928

**Published:** 2025-09-13

**Authors:** Sunghwan Shin, Dong Hee Whang, Tae-Hyun Um, Chong Rae Cho, Jeonghyun Chang

**Affiliations:** Department of Laboratory Medicine, Inje University Ilsan Paik Hospital, Goyang 10380, Republic of Koreauthmd@paik.ac.kr (T.-H.U.);

**Keywords:** Group B streptococci, *Streptococcus agalactiae*, penicillin resistance

## Abstract

**Background/Objectives**: *Streptococcus agalactiae* (Group B Streptococci, GBS) is Gram-positive, beta-hemolytic coccus known to be transmitted by vertical transmission in neonates during birth with neonatal sepsis, pneumonia, and meningitis. In adults, particularly the elderly and those with diabetes mellitus, GBS can also cause pneumonia and sepsis. Penicillin is the drug of choice, and GBS is generally susceptible to this antibiotic. This study investigates trends in GBS isolation rates and penicillin non-susceptibility over time at a university hospital. **Methods**: We retrospectively analyzed 24 years (2000–2023) of microbiological data from Ilsan Paik Hospital to investigate trends in GBS isolation and penicillin susceptibility. Isolates were identified and tested using the Vitek 2 system, following CLSI guidelines. WHONET 2023 was used for data aggregation and analysis. Trends were analyzed by dividing the study period into three intervals: Period 1 (2000–2009), Period 2 (2010–2019), and Period 3 (2020–2023). Antimicrobial susceptibility rates for total GBS and PCN-NS GBS (penicillin non-susceptible group B Streptococcus) were compared using chi-square tests. **Results**: Among 257,884 total isolates, 3003 (1.16%) were GBS, and 29 (0.97%) were PCN-NS. GBS and PCN-NS isolation rates increased significantly across the three periods (*p* = 0.0001 and *p* = 0.009, respectively). PCN-NS GBS showed reduced susceptibility to all tested antimicrobials, with no drug showing higher susceptibility compared to total GBS. **Conclusions**: This study demonstrates a statistically significant rise in both GBS isolation rate and penicillin non-susceptibility over time. Given the emergence of multidrug-resistant GBS strains, susceptibility testing and interdisciplinary collaboration between microbiologists and clinicians are critical to guiding effective antimicrobial therapy and preventing neonatal and adult GBS infections.

## 1. Introduction

*Streptococcus agalactiae* (Group B Streptococci, GBS) is a Gram-positive, beta-hemolytic coccus that tends to form chains [1]. It was first described and differentiated from other streptococci by Rebecca Lancefield in 1933 [2]. Since the first human infection of neonatal sepsis in 1964 [3], it has been well known to cause neonatal sepsis, pneumonia, and meningitis through vertical transmission from colonized mothers during delivery [4]. Maternal colonization remains common worldwide, with prevalence rates ranging from 10% to 30% depending on the region [5]. In addition to neonatal disease, GBS has emerged as an important pathogen in adults, particularly in the elderly and in individuals with comorbidities such as diabetes mellitus or malignancy, where it can cause invasive infections including bacteremia, pneumonia, and soft-tissue infections [6,7,8,9]. Population-based studies from the United States and Europe have documented a rising burden of invasive GBS disease in non-pregnant adults over the past three decades [10,11].

Beta-lactam antibiotics, especially penicillin, are the first-line treatment for GBS infections, and susceptibility to penicillin has historically been nearly universal [12]. In Clinical and Laboratory Standards Institute (CLSI) guidelines, only susceptible criteria for *S. agalactiae* are described, which is a minimum inhibitory concentration (MIC), the same as or less than 0.12 μg/mL, or more than 24 mm by disk diffusion [13]. For decades, this consistent susceptibility profile supported the clinical reliability of penicillin as a first-line agent. However, since the first report of penicillin non-susceptible (PCN-NS) GBS in 1994 [14], sporadic clinical isolates with reduced susceptibility have been documented in several countries [15,16,17]. Although the prevalence remains low, the detection of such isolates has raised concern about the potential erosion of a long-standing therapeutic standard.

Furthermore, the epidemiology of GBS itself has been changing. Several population-based studies have shown increasing isolation rates of GBS from both invasive and colonization sites, and there is accumulating evidence that the burden of adult GBS infections is rising globally [7,8,9,10]. A 2025 global meta-analysis summarizing data from 57 countries reported tetracycline resistance of over 75% and erythromycin resistance exceeding 30%, though penicillin resistance remained uncommon at 1.4%. These shifts highlight the importance of continuous local and international surveillance not only for the incidence of GBS infections but also for their antimicrobial resistance patterns [9,11].

Despite these concerns, long-term data assessing trends in penicillin susceptibility remain scarce, and most reports have been limited to short study periods or a small numbers of isolates [14]. In this study, we examined all GBS isolates recovered at a single university hospital between 2000 and 2023. Our objectives were to assess temporal trends in GBS isolation, to evaluate the occurrence of penicillin non-susceptibility, to compare antimicrobial susceptibility patterns between PCN-NS and total GBS isolates, and to analyze differences according to specimen sources. By presenting data spanning more than two decades, this study aims to contribute to a better understanding of the evolving epidemiology of GBS and to provide baseline information for future research on mechanisms of resistance and clinical management strategies.

## 2. Results

### 2.1. Study Isolates and PCN-NS Rates

From 2000 to 2023, a total of 257,884 isolates were identified and reported by our clinical microbiology laboratory. Among these, 3003 (1.16%) were identified as GBS. Within this group, 29 isolates (0.97% of GBS) were determined to be penicillin non-susceptible (PCN-NS).

### 2.2. GBS Isolation Rate and PCN-NS Trends by Period

**Figure 1:**
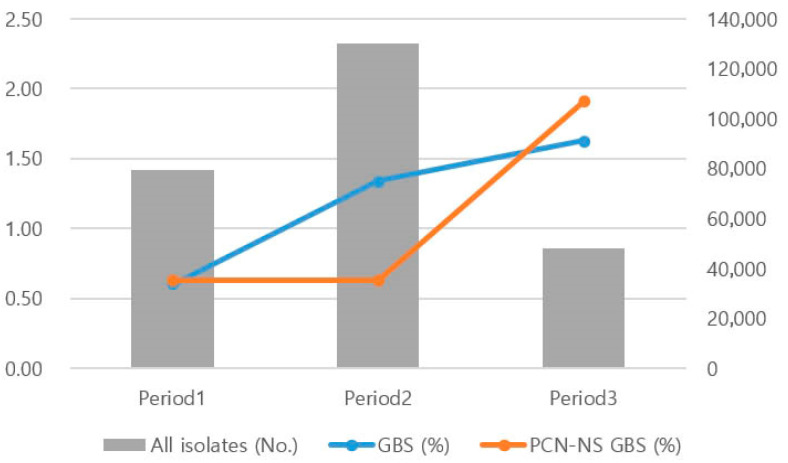
Number of isolates and percentages of *Streptococcus agalactiae* and penicillin non-susceptible *S. agalactiae* rates by period. Abbreviation: GBS: group B streptococcus, PCN-NS GBS: penicillin non-susceptible group B streptococcus.

The total number of isolates in the clinical microbiology laboratory was 79,641 during Period 1 (2000–2009), 130,108 during Period 2 (2010–2019), and 48,135 during Period 3 (2020–2023). The number of GBS isolates was 477 (0.60%) in Period 1, 1740 (1.34%) in Period 2, and 786 (1.63%) in Period 3. This demonstrated a statistically significant increasing trend over time (*p* = 0.0001). The numbers of PCN-NS GBS isolates were 3/477 (0.63%) in Period 1, 11/1740 (0.63%) in Period 2, and 15/786 (1.91%) in Period 3. This also demonstrated a statistically significant increasing trend across the study periods (*p* = 0.009) (Figure 1). Detailed yearly numbers of GBS and PCN-NS GBS isolates are provided in Supplementary Table S1.

### 2.3. Antimicrobial Susceptibility of PCN-NS GBS

Among the tested antimicrobials, tetracycline exhibited the lowest susceptibility rate in both total GBS (43.57%) and PCN-NS GBS (7.41%). In total GBS, erythromycin (62.99%), clindamycin (68.66%), and levofloxacin (76.46%) followed as the next lowest. Cefotaxime demonstrated the highest susceptibility in total GBS, with a rate of 99.86%.

In PCN-NS GBS, following tetracycline, the lowest susceptibility rates were observed for ampicillin (37.93%), erythromycin (48.28%), levofloxacin (54.55%), and clindamycin (56.00%). Cefotaxime again showed the highest susceptibility in PCN-NS GBS (90.48%). The minimum inhibitory concentration distribution of the PCN-NS isolates is provided in the Supplementary table (Table S2).

The antimicrobial susceptibility rates (%) for total GBS and PCN-NS GBS were as follows: ampicillin, 99.39/37.93 (*p* < 0.0001); ceftriaxone, 99.63/68.18 (*p* < 0.0001); cefotaxime, 99.86/90.48 (*p* < 0.0001); levofloxacin, 76.46/54.55 (*p* = 0.016); clindamycin, 68.66/56.00 (*p* = 0.175); erythromycin, 62.99/48.28 (*p* = 0.103); and tetracycline, 43.57/7.41 (*p* < 0.0001) (Table 1).

All analyzed antimicrobials demonstrated higher susceptibility rates in total GBS compared to PCN-NS GBS. There were no antimicrobials for which PCN-NS GBS exhibited higher susceptibility than total GBS.

### 2.4. Number of GBS Isolates and Penicillin Non-Susceptibility by Specimen Type

GBS isolates were recovered from 279 blood cultures, 770 vaginal swabs, 1378 urine samples, 88 respiratory specimens, and 488 other specimens. The rates of PCN-NS GBS by specimen type were as follows: 1.43% (4/279) for blood, 0.52% (4/770) for vaginal swab, 0.87% (12/1378) for urine, 3.41% (3/88) for respiratory specimens, and 1.23% (6/488) for other specimens.

The overall PCN-NS GBS rate across specimen types was 0.97% (29/3003). There was no statistically significant difference in the PCN-NS GBS rates among specimen types (*p* = 0.094) (Table 2). Although the differences were not statistically significant, isolates from respiratory specimens showed the highest rate of penicillin non-susceptibility (3.41%).

## 3. Discussion

This study evaluates the isolation rates of GBS and the trend in PCN-NS GBS over a 24-year period at a single university hospital. To our knowledge, no prior research has examined GBS isolation or PCN-NS isolation rate over such an extended timeframe in a consistent patient population. When assessing strain isolation rate or antimicrobial resistance rates, comparisons within a homogeneous patient cohort are critical to minimizing bias. The fact that this study focuses solely on isolates collected from one institution—where the patient demographic is presumed relatively stable—adds significant value to the findings. Notably, a comparable long-term analysis was recently conducted at a large tertiary hospital in Beijing, examining GBS isolates and antimicrobial resistance patterns from 2013 to 2023 [18]. However, our 24-year study remains the first of its kind to evaluate penicillin non-susceptibility trends in GBS over such an extensive duration within a single cohort, underscoring both its novelty and importance for epidemiological surveillance.

Over the 24-year period, 29 PCN-NS GBS isolates were identified in our study. Due to the low yearly counts, we divided the timeline into three 10-year periods (Period 1, 2, and 3) to analyze GBS isolation rates and PCN-NS trends. Both the GBS isolation rate and the proportion of PCN-NS GBS showed a statistically significant increase, indicating a clear upward trend over time. Such a trend has also been observed in studies conducted outside of Korea. A 2024 study from China reported an increasing detection rate of GBS from vaginal swab specimens over a 10-year observation period [18]. In the recent global meta-analysis, the prevalence of PCN-NS GBS was reported as 1.4%, which was slightly higher than the rate observed in our study (0.97%). For other antimicrobials, the reported resistance rates were 4.0% for ampicillin, 2.4% for ceftriaxone, 5.6% for cefotaxime, 8.1% for levofloxacin, 27.9% for clindamycin, 32.4% for erythromycin, and 76.7% for tetracycline [11]. Compared with our data, resistance rates to clindamycin, erythromycin, levofloxacin, and tetracycline higher, whereas resistance rates to ampicillin, ceftriaxone, and cefotaxime were higher in the global dataset. Importantly, one consistent finding between our study and the global meta-analysis is that tetracycline resistance was the highest among all tested antimicrobials. This concordance suggests that tetracycline resistance is widespread and stable across different geographic regions and study populations, further reinforcing the limited clinical utility of this agent against GBS. Since the first report of penicillin non-susceptible GBS (PCN-NS GBS) in 1994 [15], clinical isolates of PCN-NS GBS have been identified in multiple regions worldwide, including Hong Kong, Japan, Germany, and Vietnam [16,17,19,20]. In Korea, two cases of PCN-NS GBS were reported in 2019 at a single hospital, attributed to amino acid substitutions in penicillin-binding protein 2X [21].

Although the difference was not statistically significant, our study also showed the highest penicillin non-susceptibility rate among GBS isolates from respiratory specimens. Given the growing clinical significance of pneumonia, this finding warrants careful attention. Globally, several cases of pneumonia caused by PCN-NS GBS have been reported [12,22]. Pneumonia can pose serious clinical risks, especially in elderly or immunocompromised patients, necessitating heightened vigilance. Notably, our susceptibility testing showed that PCN-NS GBS isolates often exhibit multidrug resistance. In our study, these isolates were resistant not only to penicillin but also to multiple other classes of antimicrobials. This underscores the importance of cautious antibiotic selection in treatment. A study conducted in Vietnam among pregnant women reported resistance rates of 89.66% for tetracycline and 76.2% for erythromycin in GBS isolates, emphasizing the need for ongoing surveillance and resistance monitoring [20]. Furthermore, a 2017 global study reported that from 1990 to 2017, the incidence of GBS more than doubled across all age groups, with the most pronounced increase observed in the elderly population aged 65–79 years [23]. The reasons include the absence of a commercially available vaccine and the rapid acquisition of multidrug resistance [24,25].

In our study, PCN-NS GBS was also isolated from vaginal swab specimens. Vaginal swabs are of particular importance because GBS can cause neonatal meningitis through vertical transmission during delivery. Neonatal meningitis occurring during childbirth can lead to severe sequelae, including neonatal death. However, this can be effectively prevented by intrapartum antibiotic prophylaxis, and currently, pregnant women colonized with GBS routinely receive intrapartum penicillin to prevent neonatal infection [5,26]. Given our findings of PCN-NS GBS (even in vaginal isolates), it may be necessary to routinely verify penicillin susceptibility. If resistance is detected, alternative antibiotics should be used for intrapartum prophylaxis. Additionally, considering the possibility of multidrug-resistant strains [19,27], communication between infectious disease specialists and clinical microbiology experts will become increasingly important.

An important limitation of our study is the low frequency of PCN-NS GBS (29 isolates), which limits statistical power for comparisons or regression analysis. However, the strength of our study lies in the long-term, single-center dataset, which minimizes variability in methods and population, allowing a clear view of temporal trends. In addition, because the original isolates were not preserved, molecular characterization such as whole-genome sequencing could not be performed. This restriction prevented us from confirming the underlying genetic mechanisms responsible for penicillin non-susceptibility. Future investigations incorporating systematic isolate preservation and molecular analyses will be essential to provide deeper insights into the evolution of resistance in GBS. Another limitation is the absence of demographic and clinical outcome data, as this study was based on retrospective laboratory surveillance records. This lack of patient-level information restricted our ability to evaluate the real-world clinical impact of PCN-NS GBS infections.

## 4. Materials and Methods

### 4.1. Study Isolates

Isolates collected from the clinical microbiology laboratory at Ilsan Paik Hospital over a 24-year period (2000–2023) were included. Ilsan Paik Hospital is a 730-bed secondary care university hospital in a metropolitan area of South Korea. Identification and antimicrobial susceptibility testing were performed using the Vitek 2 automated system (bioMérieux, Marcy-l’Étoile, France) in accordance with the CLSI M100 guidelines [13]. Any GBS isolate with MIC > 0.12 μg/mL (or zone < 24 mm) was considered penicillin non-susceptible.

Microbial susceptibility data were analyzed using WHONET 2023 software (https://whonet.org/ (accessed on 1 August 2025)), a freely available tool developed by the World Health Organization for the management and surveillance of microbiology laboratory data. Within WHONET, the “Isolate Listing” function was applied to first classify group B Streptococcus (GBS) isolates. Subsequently, the condition of penicillin non-susceptibility was selected to generate a list of PCN-NS isolates. Data files were organized on an annual basis, which allowed us to analyze the entire 30-year dataset as well as subsets divided into three 10-year intervals. The total number of isolates, the proportion of GBS isolates, and the proportion of penicillin non-susceptible (PCN-NS) GBS were calculated over the 24-year period.

### 4.2. Division of the Study Period into Periods 1, 2, and 3

The 24-year study period was divided into three intervals: 2000–2009 (Period 1), 2010–2019 (Period 2), and 2020–2023 (Period 3). This division allowed us to evaluate long-term temporal trends and to detect potential shifts in the epidemiology of *GBS* over time. For each period, the total number of isolates, the percentage of GBS isolates, and the percentage of PCN-NS GBS were calculated. This approach enabled the assessment of both the overall burden of GBS infections and the relative increase in penicillin non-susceptibility in different decades, thereby providing insight into the dynamics of antimicrobial resistance across an extended surveillance window. In addition, the yearly numbers of GBS and PCN-NS GBS isolates are presented in Supplementary Table S1 to provide a complete view of the dataset.

### 4.3. Comparison of Antimicrobial Resistance Between PCN-NS GBS and Total GBS

Antimicrobial susceptibility rates between PCN-NS GBS and total GBS were compared in order to characterize differences in resistance profiles. The antibiotics analyzed included penicillin, ampicillin, ceftriaxone, cefotaxime, levofloxacin, clindamycin, erythromycin, and tetracycline, which represent the major drug classes clinically used for GBS infections.

### 4.4. Analysis of Antimicrobial Resistance in PCN-NS GBS Across Different Specimen Types

GBS isolates were analyzed according to specimen type in order to better understand the clinical contexts in which both total and PCN-NS GBS were recovered. Specimen types were categorized as blood, vaginal swab, respiratory specimens, urine, and others. Respiratory specimens included sputum, bronchial washings, tracheal aspirates, and throat swabs. For each category, the absolute number of isolates and the corresponding proportion of PCN-NS isolates were calculated.

### 4.5. Statistical Analysis

Statistical analysis was performed using PASW Statistics 18 software (SPSS Inc., Chicago, IL, USA). A linear-by-linear association test was used to analyze trends in GBS isolation and PCN-NS GBS rates across the defined periods. Pearson’s chi-square test was applied to compare PCN-NS rates by specimen type and antimicrobial susceptibility rates. A *p*-value of < 0.05 was considered statistically significant.

## 5. Conclusions

This 24-year retrospective study provides valuable insight into the long-term trends of GBS isolation and PCN-NS GBS within a single medical institution. Our findings revealed a statistically significant increase over time in both GBS isolation rates and the proportion of PCN-NS GBS, highlighting a growing concern for antimicrobial resistance. Although penicillin remains the mainstay of prophylaxis and treatment, the emergence of PCN-NS GBS, especially in clinically critical specimen types such as respiratory and vaginal swabs, emphasizes the need for routine susceptibility testing and careful antibiotic selection. The observed multidrug resistance patterns further underscore the importance of coordinated efforts between clinicians, infectious disease specialists, and microbiologists to ensure optimal patient care. Continued surveillance and antimicrobial stewardship will be essential in mitigating the clinical impact of resistant GBS strains, especially in vulnerable populations such as neonates, the elderly, and immunocompromised patients.

## Figures and Tables

**Table 1:** Comparison of antimicrobial susceptibility rates between GBS and PCN-NS GBS.

Antibiotics	Susceptibility Rate (%)		*p* Value
	GBS	PCN-NS GBS	
Ampicillin	99.39	37.93	<0.0001
Ceftriaxone	99.63	68.18	<0.0001
Cefotaxime	99.86	90.48	<0.0001
Levofloxacin	76.46	54.55	0.016
Clindamycin	68.66	56.00	0.175
Erythromycin	62.99	48.28	0.103
Tetracycline	43.57	7.41	<0.0001

Abbreviation: GBS: group B streptococcus, PCN-NS GBS: penicillin non-susceptible group B streptococcus.

**Table 2:** Number of *Streptococcus agalactiae* and penicillin non-susceptible *S. agalactiae* rates by specimen type.

Specimen Type	No. of GBS	No. of PCN-NS GBS	PCN-NS Rate (%)
Blood	279	4	1.43
Vaginal swab	770	4	0.52
Urine	1378	12	0.87
Respiratory specimen	88	3	3.41
Others	488	6	1.23
Total	3003	29	0.97

Abbreviation: GBS: group B streptococcus, PCN-NS GBS: penicillin non-susceptible group B streptococcus.

## Data Availability

The data presented in this study are available on request from the corresponding author. The data are not publicly available because it contains internal information about the medical institution.

## Supplementary Materials

The following supporting information can be downloaded at: https://www.mdpi.com/article/10.3390/antibiotics14090928/s1, Table S1: Yearly numbers of *Streptococcus agalactiae* and penicillin non-susceptible *Streptococcus agalactiae* isolates (2000–2023). Table S2: Minimum inhibitory concentration distribution of 29 penicillin non-susceptible isolates.

## Abbreviations

The following abbreviations are used in this manuscript:

GBS	group B streptococcus
PCN-NS GBS	penicillin non-susceptible group B streptococcus
