# Supplementary material for: Increase in Penicillin Non-Susceptibility in Group B Streptococci Alongside Rising Isolation Rates—Based on 24 Years of Clinical Data from a Single University Hospital
Source: Antibiotics (Basel). 2025 Sep 13;14(9):928. doi: 10.3390/antibiotics14090928 (PMC12466618; doi:10.3390/antibiotics14090928)
Supplement: Supplementary file 1 [file antibiotics-14-00928-s001.zip › antibiotics-3858628-supplementary.pdf]

Table S1. Yearly numbers of *Streptococcus agalactiae* and penicillin non-susceptible *Streptococcus agalactiae* isolates (2000–2023)

Year	No. of GBS	No. of PCN-NS GBS	Total No. of specimens
2000	13	1	1583
2001	41	0	5265
2002	19	0	6733
2003	39	0	7389
2004	54	0	8081
2005	71	0	10340
2006	48	1	9654
2007	70	0	10102
2008	69	1	10018
2009	53	0	10476
2010	79	1	11048
2011	73	0	12843
2012	133	3	13383
2013	139	0	12910
2014	196	0	14872
2015	229	1	13190
2016	258	6	13263
2017	226	0	12401
2018	205	0	13691
2019	202	0	12507
2020	193	4	11951
2021	210	6	11744
2022	219	4	12043
2023	164	1	12397

Abbreviation; GBS: group B streptococcus, PCN-NS GBS: penicillin non-susceptible group B streptococcus.

Table S2. Minimum inhibitory concentration distribution of 29 penicillin non-susceptible isolates

Isolate ID	Minimal inhibitory concentration (μg/mL)

	Ampicillin	Cetotaxime	Ceftriaxone	Levofloxacin	Clindamycin	Erythromycin	Tetracycline
01	2	N/A	N/A	N/A	> 4	2	> 8
02	> 8	N/A	N/A	N/A	> 4	> 4	N/A
03	2	N/A	N/A	N/A	≤ 0.25	≤ 0.25	≤ 1
04	≤ 0.25	N/A	N/A	N/A	N/A	> 4	N/A
05	2	N/A	N/A	N/A	N/A	≤ 0.25	> 8
06	2	N/A	N/A	N/A	N/A	≤ 0.25	> 8
07	2	N/A	N/A	N/A	N/A	≤ 0.25	≤ 1
08	2	≤ 0.12	≤ 0.12	> 8	> 0.5	> 4	> 8
09	2	N/A	≤ 0.12	> 8	> 0.5	> 4	> 8
10	2	≤ 0.12	≤ 0.12	1	≤ 0.25	≤ 0.12	> 8
11	2	≤ 0.12	≤ 0.12	0.5	≤ 0.25	2	> 8
12	2	≤ 0.12	≤ 0.12	1	≤ 0.25	≤ 0.12	> 8
13	2	≤ 0.12	≤ 0.12	1	≤ 0.25	≤ 0.12	> 8
14	2	≤ 0.12	≤ 0.12	1	≤ 0.25	≤ 0.12	> 8
15	≤ 0.25	≤ 0.12	≤ 0.12	0.5	≤ 0.25	> 4	> 8
16	≤ 0.25	0.5	1	> 8	> 0.5	> 4	> 8
17	≤ 0.25	0.5	1	> 8	> 0.5	> 4	> 8
18	≤ 0.25	≤ 0.12	1	0.5	≤ 0.25	2	> 8
19	≤ 0.25	≤ 0.12	0.25	> 8	≤ 0.25	≤ 0.12	> 8
20	≤ 0.25	≤ 0.12	0.25	> 8	≤ 0.25	≤ 0.12	> 8
21	1	2	4	1	> 0.5	> 4	> 8
22	≤ 0.25	0.5	0.5	> 8	> 0.5	> 4	> 8
23	≤ 0.25	0.5	1	> 8	> 0.5	> 4	> 8
24	≤ 0.25	0.5	1	> 8	> 0.5	> 4	> 8
25	2	≤ 0.12	≤ 0.12	0.5	≤ 0.25	≤ 0.12	> 8
26	2	≤ 0.12	≤ 0.12	1	≤ 0.25	≤ 0.12	> 8
27	2	≤ 0.12	≤ 0.12	1	≤ 0.25	≤ 0.12	> 8
28	≤ 0.25	4	> 4	> 8	> 0.5	2	> 8
29	2	≤ 0.12	≤ 0.12	1	≤ 0.25	≤ 0.12	> 8

Abbreviation; N/A, not available
